# Electrospun PVA/Co_3_O_4_ Nanofibers: A Sustainable Catalyst for Peroxymonosulfate-Mediated Degradation of Tetracycline

**DOI:** 10.1021/acsomega.5c01013

**Published:** 2025-06-25

**Authors:** Felipe G. Kirchhoff, Gabriel N. Fraga, Reinaldo A. Bariccatti, Douglas C. Dragunski, Guilherme G. Bessegato

**Affiliations:** † 201362 Universidade Estadual do Oeste do Paraná (UNIOESTE), Rua da Faculdade 645, 85903-000 Toledo, Paraná, Brazil; ‡ Department of Chemistry, Universidade Estadual de Maringá (UEM), Av. Colombo, 5790—Zona 7, 87020-900 Maringá, Paraná, Brazil; § Universidade Tecnológica Federal do Paraná (UTFPR), Dois Vizinhos Campus, Estrada para Boa Esperança, km 04, 85660-000 Dois Vizinhos, Paraná, Brazil

## Abstract

This study develops a poly­(vinyl alcohol) (PVA) nanofiber incorporated with cobalt oxide (Co_3_O_4_) for catalytic degradation of antibiotics using peroxymonosulfate (PMS) to generate sulfate radical (SO_4_^•–^) (*E*° = 2.5–3.1 V *vs* NHE). These radicals efficiently degrade tetracycline through selective oxidation, offering advantages over conventional hydroxyl radical-based processes. The nanofiber fabrication involved electrospinning of PVA (8% w/v) containing cobalt oxide suspension (5 g L^–1^) and citric acid (15% w/w polymer). Thermal cross-linking at 160 °C for 2 h enhanced the material’s aqueous stability and mechanical properties. Scanning electron microscopy revealed uniform fibers (627–645 nm diameter), while X-ray diffraction, thermal analyses, and spectroscopy confirmed increased crystallinity in PVA/Co_3_O_4_ composites. Optimization studies through factorial design identified pH and PMS concentration as key parameters, achieving 60% tetracycline degradation within 60 min. The successful integration of Co_3_O_4_ into biodegradable PVA nanofibers presents a sustainable, cost-effective approach for water treatment applications, particularly targeting emerging pharmaceutical contaminants.

## Graphical abstract

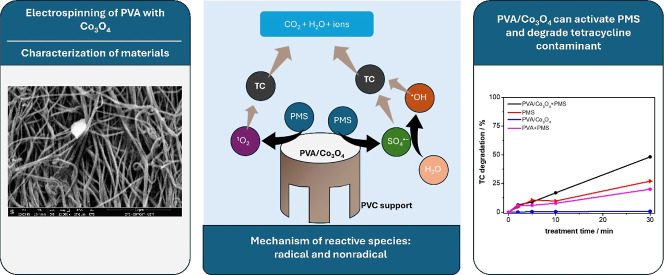

## Introduction

Continuous population growth has led to a significant increase in surface and underground water pollution. Emerging contaminants such as pharmaceuticals, agricultural pesticides, and personal care products persist in the environment due to the lack of systematic risk assessments despite the proven toxicity of these compounds. These compounds have been proven toxic and pose serious risks to environmental ecosystems and human health. They can disrupt aquatic life, contribute to antibiotic resistance, and have been linked to various adverse health effects in humans, including endocrine disruption and increased cancer risk.^1,2^

Among the contaminants of emerging concern, antibiotics are of particular significance. Widely used in human and veterinary medicine, antibiotics frequently enter water bodies through wastewater discharge and agricultural runoff.^3^ These compounds can promote the development of antibiotic-resistant bacteria and disrupt microbial communities essential for nutrient cycling and ecosystem stability. Antibiotic contamination in water can spread resistance genes, posing a significant threat to public health by reducing the efficacy of essential antibiotics.^3,4^ One antibiotic of significant concern is tetracycline. Extensively used due to its broad-spectrum activity, tetracycline is commonly detected in water environments. Its presence poses several environmental issues, including promoting antibiotic-resistant bacteria and disrupting aquatic microbial communities. For humans, exposure to tetracycline through contaminated water can lead to allergic reactions, gastrointestinal disturbances, and the proliferation of antibiotic-resistant pathogens, posing significant public health risks.^5,6^

Advanced oxidation processes (AOPs) have proven effective in degrading these recalcitrant contaminants in water, air, and soil. AOPs offer several advantages, including faster reaction rates compared to conventional treatment technologies, and the ability to degrade small and highly toxic molecules such as pharmaceuticals and pesticides.^7,8^ Examples of AOPs include peroxide- and ozone/ultraviolet-based processes, electrochemical oxidation treatments, and advanced catalytic oxidation.^7,9^

Among these methods, persulfate activation has gained attention as a promising strategy. This process generates highly reactive sulfate radicals (SO_4_^•–^) via catalytic or photoactivated persulfate activation.^10,11^ These radicals possess exceptional oxidative potential (2.5–3.1 V vs NHE), enabling the efficient degradation of various organic pollutants.^12–14^ One such persulfate is peroxymonosulfate (HSO_5_^–^), commercially known as Oxone. It can be converted into SO_4_^•–^ radicals via electron transfer when reacted with transition metal ions such as Mn^2+^, Ce^3+^, Ni^2+^, Fe^2+^, V^3+^, Ru^3+^, and Co^2+^. Systems based on Co^2+^/PMS are advantageous. Recent studies have highlighted the development of heterogeneous cobalt-based systems that operate efficiently in neutral pH conditions.^15^ Furthermore, even with low catalyst concentrations, persulfate activation has demonstrated significant potential, yielding satisfactory results in pollutant degradation.^16–18^

However, reusing catalysts, particularly Co^2+^, raises environmental concerns due to their potentially harmful effects. An alternative approach involves supporting the catalyst on a substrate. Recently, studies have demonstrated the potential of catalysts supported on electrospun nanofibers, highlighting the advantages of incorporating metals through this technique. The high porosity of the nanofibers, combined with the formation of a composite with a high surface area, facilitates the creation of catalytic sites along the polymer chain, providing a structure capable of efficiently activating persulfates. Furthermore, cobalt oxides have been successfully incorporated into polymers using electrospinning, offering promising catalytic properties for environmental applications.^19,20^ However, nonbiodegradable polymers have been used, posing environmental issues, high costs, and the need for organic solvents.^21–23^ Poly­(vinyl alcohol) (PVA) presents a viable solution characterized by its water solubility, biodegradability, and low cost. PVA can be electrospun without organic solvents, using water exclusively. Additionally, its solubility can be reduced through cross-linking, providing the resulting nonwoven fabric with resistance to degradation cycles.^24^

This study presents a novel approach by investigating the potential of generating sulfate radicals using cobalt oxide incorporated into electrospun PVA fibers. The PVA/Co_3_O_4_ material was characterized, and the efficiency of PVA/Co_3_O_4_ fibers in the catalytic activation of peroxymonosulfate for the degradation of the antibiotic tetracycline was examined, along with the mechanism of reactive species generation.

## Experimental Section

### Synthesis of Cobalt Oxide (Co_3_O_4_)

Cobalt oxide was synthesized by adding 100 mL of a 2.5 mol L^–1^ sodium hydroxide (NaOH, Synth, ≥97% purity) solution dropwise, with constant stirring, to a solution of 100 mL of 0.5 mol L^–1^ cobalt nitrate hexahydrate (Co­(NO_3_)_2_·6H_2_O, Neon, ≥98% purity). A dark-colored suspension formed, which was filtered to obtain a gray precipitate.^25^ This precipitate was dried in an oven for 3 days at 60 °C and then subjected to thermal treatment at 400 °C for 2 h in an ambient atmosphere to obtain cobalt oxide (Co_3_O_4_).^26^ The reactions for obtaining cobalt oxide are shown in eqs 1 and 2:

1 $$Co{\left({NO}_{3}\right)}_{2}{\cdot 6H}_{2}O+2NaOH\to CoO\left(OH\right)+{2NaNO}_{3}+{6H}_{2}O$$

2 $$4CoO\left(OH\right)\to {2Co}_{2}O_{3}+{2H}_{2}O\to {2Co}_{3}O_{4}+{1/2O}_{2}$$

### Obtaining the Nanofibers

The electrospinning technique was employed to produce the nanofibers. Poly­(vinyl alcohol) (PVA) (Neon, 104.5 g mol^–1^) with a degree of hydrolysis of 87%–89% was used as the polymer. To prepare the polymeric solution, 0.8 g of PVA (8% w/v) was dissolved in 9.0 mL of preheated distilled water and stirred in a water bath at 90 °C until complete dissolution. A 1.0 mL suspension containing cobalt oxide (5 g L^–1^) in ethanol (Neon, 99.5% purity) and 0.12 g of citric acid (CA, Neon, ≥99% purity) (15% w/w relative to PVA) was prepared. The PVA solution was mixed with the cobalt oxide suspension and stirred for 30 min. Electrospinning was performed with a flow rate of 0.5 mL h^–1^, voltage of 25 kV, and a needle-to-collector distance of 15 cm, using a rotating collector at 1000 rpm. The ambient conditions during electrospinning were 20–25 °C and <50% relative humidity. After electrospinning, the PVA and PVA/Co_3_O_4_ fibers were cross-linked in an oven at 160 °C for 2 h.^24^ A standard PVA 8% sample without citric acid was also electrospun as a control.

### Characterization of Nanomaterials

The prepared fibers were analyzed using scanning electron microscopy (SEM) (FEI Quanta 250). Samples (1 cm × 1 cm) were metalized, deposited on double-sided carbon adhesive tape, and coated with gold (30 nm thickness). SEM analyses were performed at magnifications of 2000×, 5000×, 10,000×, and 15,000×. Fiber diameters were measured using ImageJ software (*n* = 100) and analyzed using Tukey’s test.

Fourier Transform Infrared (FTIR) spectra were obtained using an ATR module (PerkinElmer, Frontier model) with wavenumbers ranging from 4000 to 650 cm^–1^, 2 cm^–1^ resolution, and eight accumulations.

X-ray Diffraction (XRD) analyses were performed using Bruker D2 Phaser equipment with a scan angle from 10° to 80°, 0.02° θ increment, 30 kV voltage, 10 mA current, and Cu Kα radiation (λ = 1.5418 Å). The crystallinity was calculated using eq 3, which involves the difference between the nanomaterials’ crystalline areas and the analysis’s total areas

3 $$\frac{crystalline\;area}{Total\;área}\times 100$$

Energy dispersive X-ray fluorescence analysis was performed to identify cobalt oxide in the nanomaterials using a SHIMADZU EDX-7200 model device, operating at 100 μA current and 50 kV voltage. The nanomaterials were placed on a polypropylene support and covered with a mylar membrane.

Thermogravimetric analysis (TGA) and differential scanning calorimetry (DSC) were performed to evaluate thermal properties. TGA was performed using a PerkinElmer STA 6000 equipment with a sample mass of 6 mg, temperature range of 30 to 700 °C, heating rate of 10 °C min^–1^, and nitrogen flow rate of 50 mL min^–1^. DSC was carried out on a Shimadzu DSC 60 with a sample mass of 6 mg, temperature range of 30 to 200 °C, heating rate of 10 °C min^–1^, and nitrogen flow rate.

Mechanical properties were analyzed using a portable universal testing machine (Biopdi/Brazil). Samples (1 cm × 5 cm) were prepared per ASTM standards, and thickness was measured with an analog thickness gauge (Mitutoyo, n° 7301). In duplicate, a 5 kgf load cell at 10 mm min^–1^ displacement speed was used for tensile testing.

### Zero Point of Charge (pH_pzc_)

To determine the pH_pzc_, six 10 mL beakers containing 5 mL of 0.1 mol L^–1^ KCl solution each were prepared. The pH of each solution was adjusted from 3 to 8 using diluted HCl and NaOH solutions (0.1 mol L^–1^). A 1 cm^2^ piece of PVA/Co_3_O_4_ nonwoven fabric was added to each beaker. After 24 h, the pH was measured again. An initial pH versus final pH graph was constructed to determine the pH_pzc_, including the standard PVA nonwoven fabric.

### Degradation of a Model Contaminant by PMS Catalysis

A 2^2^ factorial experimental design with a central point was used to evaluate the degradation of a 16.8 mg L^–1^ tetracycline hydrochloride solution (TC, Exodo Cientifica, >97%) by activation of peroxymonosulfate (KHSO_5_·0.5KHSO_4_·0.5K_2_SO_4_, Sigma-Aldrich, 307.38 g mol^–1^). PMS concentrations and pH levels were varied, as shown in Table 1. The nonwoven fabrics were fixed on a homemade polyvinyl chloride polyvinyl (PVC) support with the help of Teflon tape. TC degradation was monitored using UV–vis spectrophotometry (Shimadzu, PC-1800) at 356 nm, with aliquots analyzed at 2, 5, 10, 30, and 60 min. Twenty-five mL of 16.8 mg L^–1^ TC solution was used for each degradation experiment, with the necessary volume of PMS stock solution added to achieve the concentrations in Table 1. Degradation was monitored at 356 nm using UV–vis spectrophotometry, with aliquots analyzed at 0, 2, 5, 10, 30, and 60 min. Each experiment was performed in triplicate to ensure reproducibility.

**Table 1:** Central Composite 2^2^ Factorial Design, Where the Variables pH and PMS Concentration Applied in the Systems are Presented, along with the Levels Used^a^

experiment	coded levels		real values	
	*x*_1_	*x*_2_	*x*_1_^a^	*x*_2_^b^
1	–1	–1	3.00	0.567
2	–1	1	3.00	1.700
3	1	–1	9.00	0.567
4	1	1	9.00	1.700
5	0	0	6.00	1.130
6	0	0	6.00	1.130
7	0	0	6.00	1.130

a x_1_^a^ = pH; x_2_^b^ = [PMS]/mmol L^–1^.

### Identification of Reactive Species Using Scavengers

TC degradation (25 mL, 16.8 mg L^–1^) was conducted in the presence of different scavengers to identify the mechanism of reactive species generation.^27^ PMS stock solution was added to achieve 1.1 mmol L^–1^ along with 5.6 × 10^–2^ mol L^–1^ of scavengers: sodium azide (NaN_3_, Neon, ≥99%), methanol (Neon, ≥99.8%), and *t*-butanol (Neon, ≥99%). The choice of scavengers was based on their ability to selectively quench specific reactive oxygen species (ROS): sodium azide for singlet oxygen,^28^ methanol for hydroxyl radicals and sulfate radicals, and *t*-butanol for hydroxyl radicals.^29^ Degradation was also monitored at 2, 5, 10, 30, and 60 min using UV–vis spectroscopy (Shimadzu, PC-1800).

### Stability of Electrospun PVA/Co_3_O_4_

The replicability of the films was assessed through degradation experiments using the model contaminant TC (16.8 mg L^–1^), with an aliquot added to a PMS concentration of 1.1 mmol L^–1^. Five experiments were conducted for degradation analysis, during which the nanomaterials were cleaned by immersing them in deionized water at 10 min intervals between degradation cycles. Aliquots were analyzed at 2, 5, 10, and 30 min using UV–vis molecular spectrophotometry (Shimadzu, PC-1800).

For further analysis, standard TC solutions (16.8 mg L^–1^) and the solutions after each degradation cycle were examined using an X-ray spectrophotometer (EDX-7200 SHIMADZU). The parameters set for the equipment included a voltage of 50 kV and an applied current of 100 μA. The solutions were placed in a polypropylene sample holder with a mylar membrane and analyzed in an air atmosphere. Rhodium (Rh) was used as the target source.

## Results and Discussion

### Materials Characterization

#### Scanning Electron Microscopy

The morphology and diameter of the PVA and PVA/Co_3_O_4_ fibers were analyzed using SEM (Figure 1). Both fibers exhibited a uniform, bead-free nanofibrous structure. The average fiber diameter for PVA fibers was 627.43 ± 183.67 nm, while that for PVA/Co_3_O_4_ fibers was slightly larger at 644.91 ± 199.78 nm. Statistical analysis (Tukey test, 95% confidence level) indicated no significant difference between the two groups, suggesting that incorporating cobalt oxide did not substantially alter the fiber formation process.^30^ Figures S1 and S3 show the SEM image and the X-ray diffractogram of the prepared oxide, respectively. The diffractogram confirms that the incorporated oxide is Co_3_O_4_, an excellent heterogeneous catalyst for PMS activation.^31^

**Figure 1:**
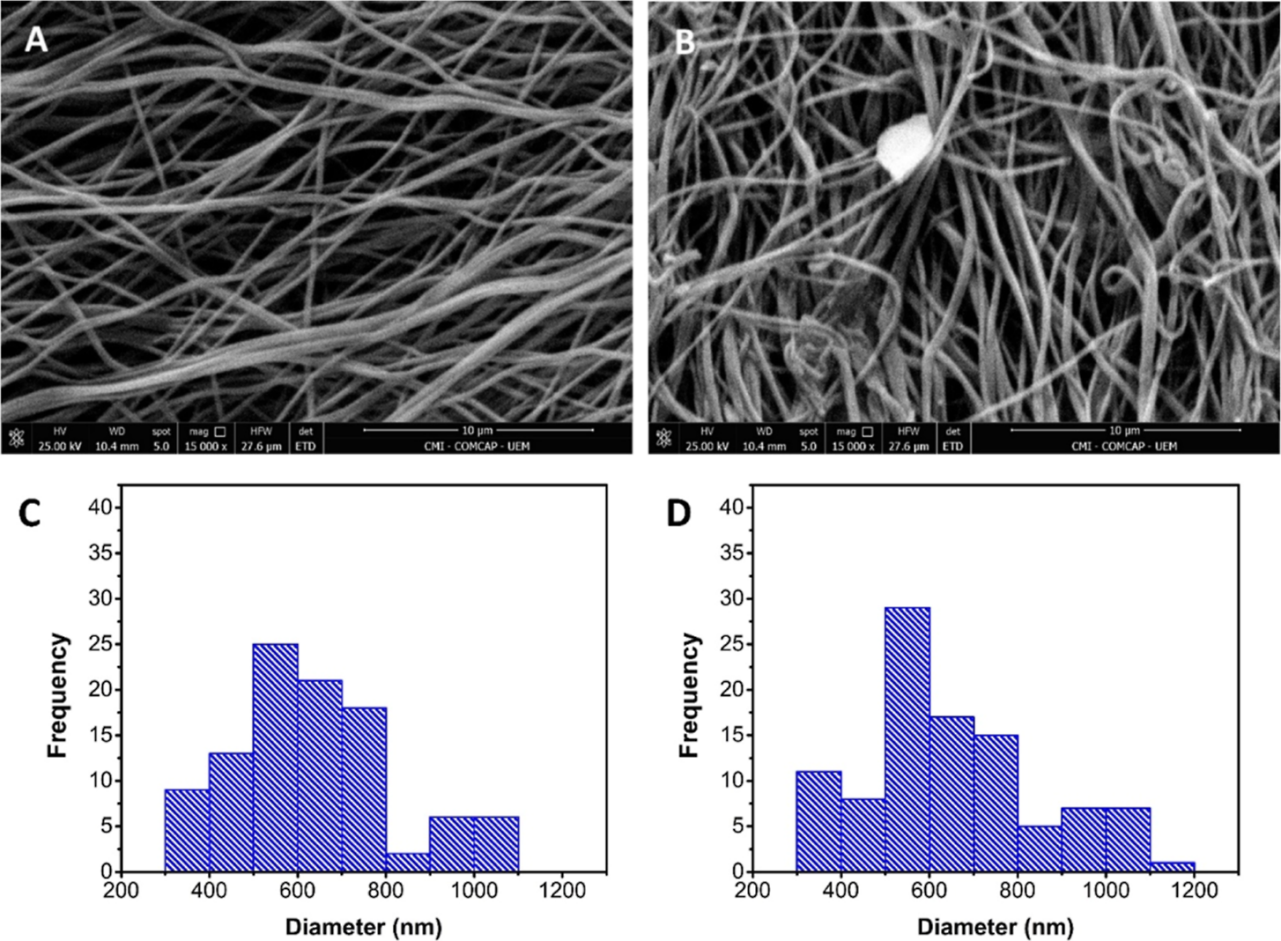
Images obtained by scanning electron microscopy (SEM) of electrospun nonwovens of PVA (A) and PVA/Co_3_O_4_ (B) at 15,000× magnification, with the average fiber diameter represented in the histogram, respectively (C,D).

#### Fourier Transform Infrared Spectroscopy

The presence of functional groups in the electrospun membranes of PVA and PVA/Co_3_O_4_ was investigated using Fourier Transform Infrared (FTIR) spectroscopy (Figure 2). For PVA, it is possible to identify the broadening of the band at 3326 cm^–1^, associated with the vibrational stretching of O–H groups of inter- and intramolecular bonds. The 2940 and 2920 cm^–1^ bands represent the symmetric and asymmetric vibrations characteristic of methylene (CH_2_) stretching groups. At 1714 cm^–1^, there are C=O bending vibrations from acetate residues from the cross-linking process. The band positioned at 1190 cm^–1^ is related to the stretching in the crystalline region of the polymer due to a symmetric vibration of the C–C bond. The band at 1090 cm^–1^ refers to the stretching of asymmetric vibrations C–O and the bending of O–H. At 844 cm^–1^, the vibration is attributed to the balance of methylene groups (CH_2_).^32^

**Figure 2:**
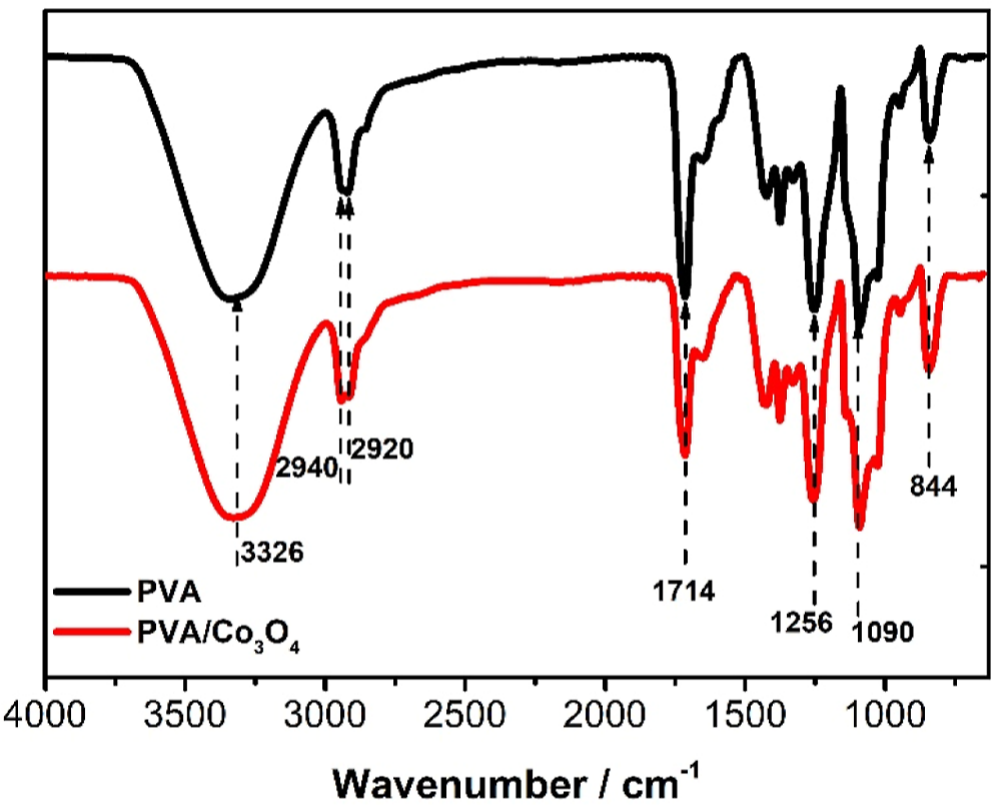
Fourier-transform infrared (FTIR) spectra using the ATR module of nonwoven fabrics of poly­(vinyl alcohol) (PVA) and PVA/Co_3_O_4_, both reticulated with citric acid.

No distinct peaks attributable to Co_3_O_4_ were observed, likely due to the low concentration of the oxide and its encapsulation within the polymer matrix. This finding is further supported by the X-ray diffraction (XRD) results (Figure 3), where characteristic peaks for Co_3_O_4_ were not detected. This observation is significant as it suggests that the oxide nanoparticles are well-dispersed throughout the nanofibers. However, EDX analyses (Figures S4 and S5) confirm the presence of cobalt in the nanofibers, indicating the existence of cobalt oxide in the nanomaterials, which complements the findings from the XRD and FTIR analyses.

**Figure 3:**
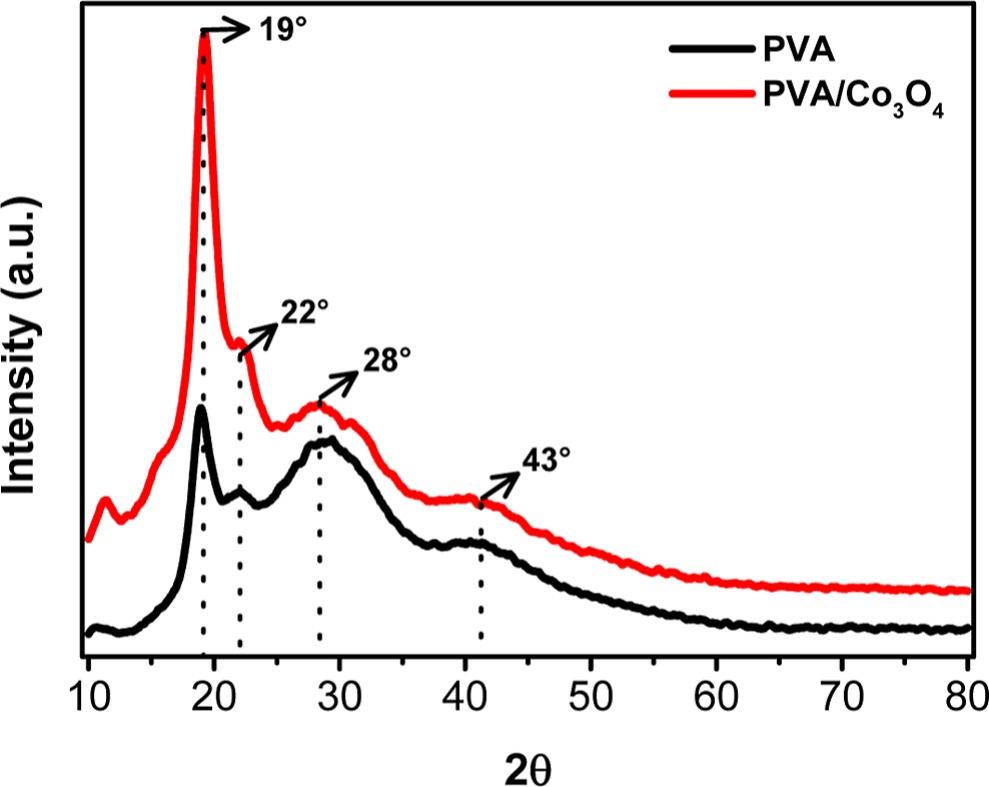
X-ray diffractograms (XRD) for PVA and PVA/Co_3_O_4_ electrospun nonwovens, both reticulated with citric acid.

#### X-ray Diffraction

The crystalline phases present in the electrospun materials were analyzed using X-ray diffraction (XRD) (Figure 3). A peak at 2θ = 19° was characteristic of the crystalline plane (101) for both PVA and PVA/Co_3_O_4_, confirming the presence of the PVA crystalline phase.^33,34^ The intensity of this peak was observed to be higher in the PVA/Co_3_O_4_ sample, suggesting an increase in crystallinity.^24^ Additionally, a peak at 22° was attributed to hydrogen bonds within the PVA crystallites. The peak at 28° corresponds to the sample support (Figure S2). The peak at 43° refers to the semicrystalline region characteristic of the polymer.^35^

The comparative analysis of peak intensities in the PVA and PVA/Co_3_O_4_ nonwoven fabrics reveals an increase in peak intensity with the insertion of Co_3_O_4_. This can be attributed to the increased crystallinity (about 70%) upon cobalt oxide incorporation. The oxygen present on the surface of the oxide interacts with the –OH groups present in the polymeric chains of PVA, leading to a more explicit definition of the polymeric crystallites.^36–38^

#### Thermogravimetric Analysis (TG)

The results of thermal stability analyses of electrospun and cross-linked PVA and PVA/Co_3_O_4_ materials are represented in Figure 4a. Between 30 to 90 °C, the first event associated with the evaporation of water molecules adsorbed on the surface of the nonwoven fabric is located, which exhibits hydrophilic characteristics. Thermal degradation can be related to the second event, between 225 and 405 °C, where the loss of –OH groups occurs, resulting in conjugated and nonconjugated polyenes. Between 415 and 510 °C, reactions related to chain cleavage occur, resulting in carbonized substrates.^39,40^

**Figure 4:**
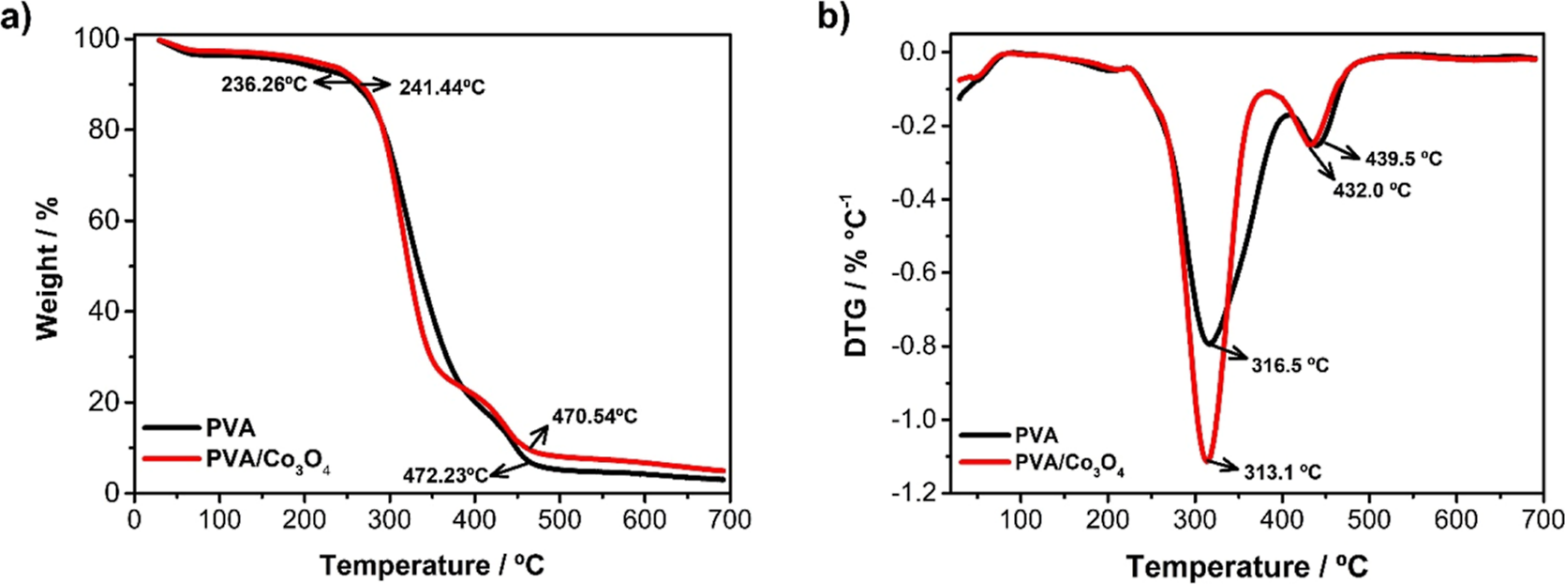
(a) Thermogravimetric analysis (TGA) curves and (b) derivative thermogravimetric analysis (DTG) curves of poly­(vinyl alcohol) nonwovens (PVA), and poly­(vinyl alcohol) incorporated with a 10% (v/v) cobalt oxide suspension nonwovens (PVA/Co_3_O_4_).

The electrospun nanomaterials of PVA/Co_3_O_4_ exhibited thermal degradation in three stages, like the cross-linked PVA. The DTG curves in Figure 4b show no significant difference in the first event for the PVA and PVA/Co_3_O_4_ materials.^39,41^ The PVA/Co_3_O_4_ nonwoven fabrics presented a decrease in thermal stability due to the increase in peak intensity and shift, accelerating the decomposition of the nanomaterial. Incorporating a material with thermal conductor characteristics may increase the nanomaterial’s thermal degradation rate.^42^

Mathematical deconvolution of DTG curves (Figures S7–S9) suggests that the incorporation of cobalt oxide may facilitate the dehydration of the material at a lower temperature, consequently lowering the onset temperature of the second degradation stage and promoting the loss of −OH groups to form conjugated and nonconjugated polyenes.

#### Differential Scanning Calorimetry

Differential scanning calorimetry analyses of the electrospun PVA and PVA/Co_3_O_4_ materials can be observed in Figure 5. Peak 1, in the range of 30 to 110 °C, is associated with water loss due to the moisture in the samples. Two peaks can be noted in the PVA/Co_3_O_4_ nanomaterial, which may be related to the polymer’s surface and internal water loss, possibly due to interactions with the cobalt oxide encapsulated in the nanomaterial.

**Figure 5:**
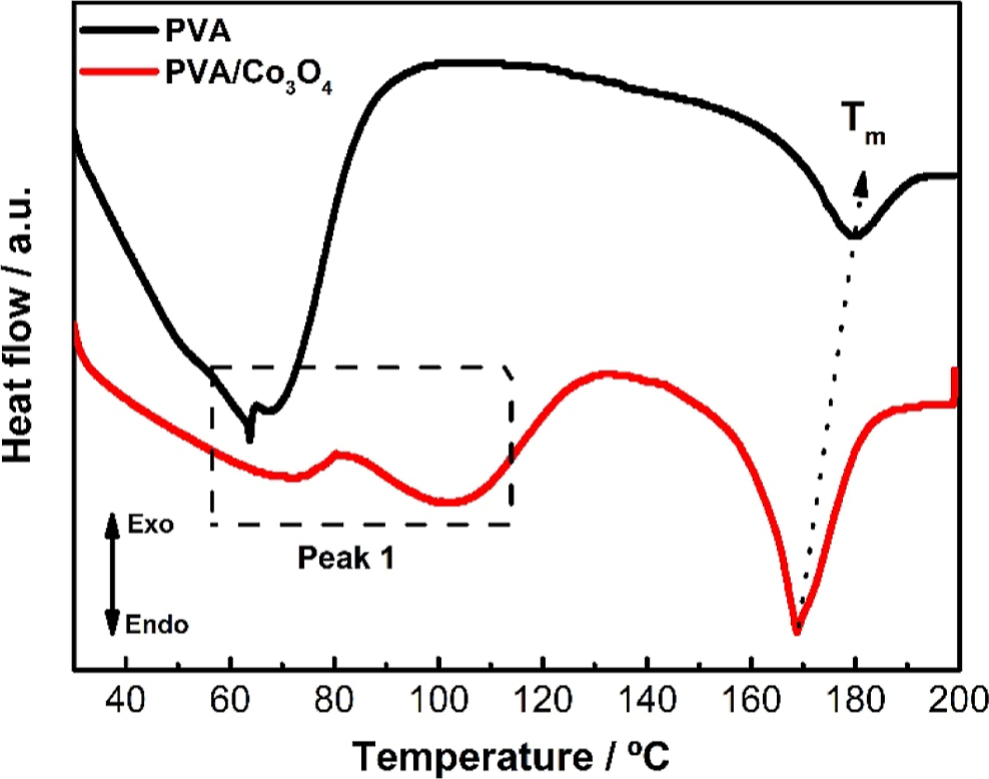
Differential scanning calorimetry (DSC) analysis of poly­(vinyl alcohol) nonwoven (PVA) and poly­(vinyl alcohol) incorporated with cobalt oxide nonwoven material (PVA/Co_3_O_4_).

The second peak (endothermic) refers to the melting phenomenon of PVA. Incorporating cobalt oxide lowers the melting point, although an increase in the enthalpy of fusion (Δ*H*) was observed (see Table 2). The decrease in the melting temperature suggests that the oxide may induce a reorganization of the polymer chains, altering the crystal structure and leading to a lower melting point, corroborating the XRD diffractograms (Figure 3).^41^

**Table 2:** Parameters Obtained from DSC Analyses^a^

sample	*T*_m_ (°C)	Δ*H*_melting_ (J g^–1^)
PVA	179.73	10.91
PVA/Co_3_O_4_	169.01	30.72

a *T*_m_—melting temperature in °C; Δ*H*_melting_—enthalpy of fusion in J g^–1^. Poly­(vinyl alcohol) nonwoven (PVA) and poly­(vinyl alcohol) incorporated with cobalt oxide nonwoven (PVA/Co_3_O_4_).

#### Mechanical Strength

The mechanical analysis for the electrospun materials is presented in Figure 6. The cross-linking implemented in the electrospun fibers results in increased stiffness and reduced flexibility, thus decreasing the elongation of the nonwoven fabric.^24^ The changes in crystallinity (confirmed by XRD and DSC) also translated to altered mechanical properties. PVA/Co_3_O_4_ fibers exhibited higher tensile strength and Young’s modulus (Table 3), indicative of a more rigid and resistant material compared to pure PVA fibers. This is consistent with the observed increase in crystallinity, as stronger intermolecular interactions within the crystalline regions would lead to greater mechanical strength.

**Figure 6:**
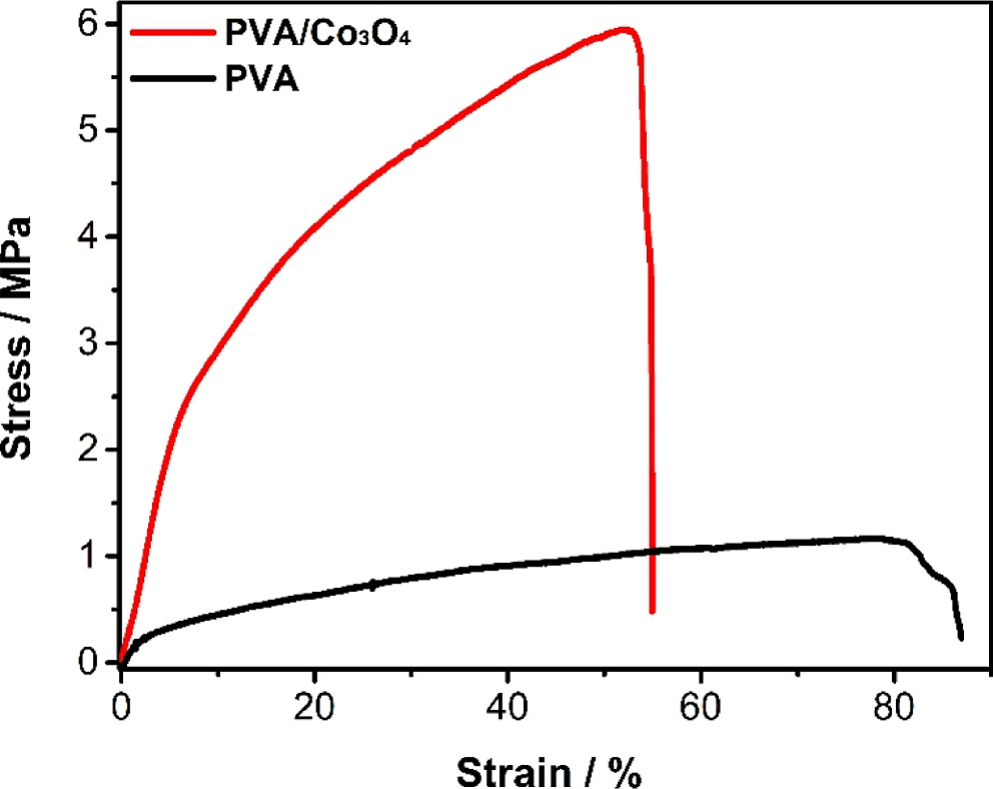
Stress–strain curves for PVA and PVA/Co_3_O_4_ nonwoven fabrics.

**Table 3:** Mechanical Strength Analyses of the Nanomaterials (Performed in Duplicates)^a^

sample	σ (MPa)	ε (%)	modulus of elasticity (MPa)
PVA	1.165 ± 0.05	67.13 ± 19.81	0.14 ± 0.02
PVA/Co_3_O_4_	6.57 ± 0.62	53.85 ± 1.09	0.56 ± 0.09

a σ—breakdown voltage in MPa; ε—stretching in %; Young’s Module—Young’s modulus in MPa.

#### Zero Point of Charge (pHpcz)

Figure 7 presents the final pH—initial pH versus initial pH graph to determine the pH_pzc_. The point where the line intersects the *x*-axis (at *y* = 0) corresponds to the pH_pzc_. For the PVA/Co_3_O_4_ sample (Figure 7b), the pH_pzc_ was determined to be 4.36, while pure PVA (Figure 7a) exhibited a zero charge point at 4.43, indicating no significant difference upon oxide incorporation. These results corroborate previous findings suggesting that the oxide is effectively encapsulated within the polymer matrix, thus not significantly altering the surface charge properties. These pH_pzc_ values indicate that the surface of the materials becomes positively charged below this point (due to protonation) and negatively charged above it (due to deprotonation).

**Figure 7:**
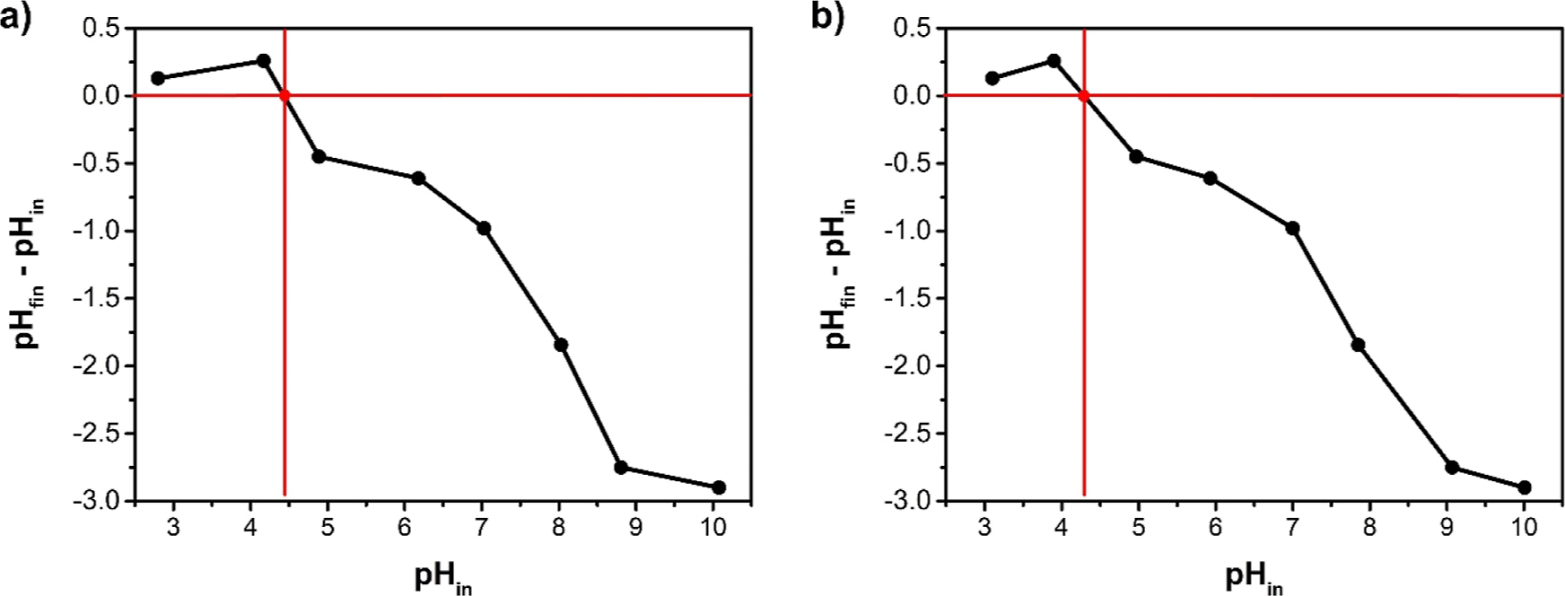
Graph of (final pH—initial pH) versus initial pH for the determination of pH_pzc_ (point of zero charge): (a) for PVA and (b) for PVA/Co_3_O_4_.

#### Degradation of the Model Contaminant: Antibiotic Tetracycline

The catalytic activity of the Co_3_O_4_-modified PVA nanofibers was evaluated through their ability to activate peroxymonosulfate (PMS) for tetracycline (TC) degradation, which served as a model antibiotic contaminant. Degradation experiments were conducted under various conditions: PVA/Co_3_O_4_ nanofibers with and without PMS, pure PVA fibers with PMS, and PMS alone (Figure 8).

**Figure 8:**
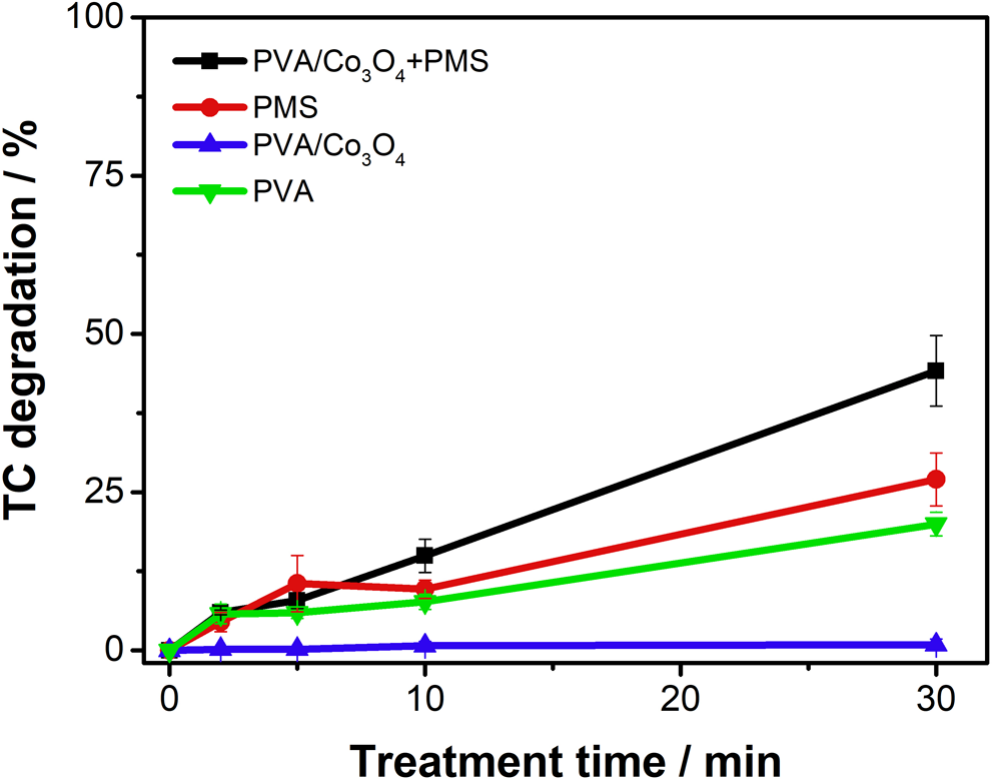
Degradation of TC versus treatment time for: (−■−) PVA/Co_3_O_4_ in the presence of PMS and (−▲−) without PMS; (−●−) just PMS, and (−▼−) PVA in the presence of PMS. Conditions:16.8 mg L^–1^ TC solution, pH 6, [PMS] = 1.1 mmol L^–1^, under constant stirring.

The results demonstrate that PVA/Co_3_O_4_ material effectively activates PMS for TC degradation, achieving 48.2% degradation efficiency after 30 min—significantly higher than the 27.0% observed with PMS alone. Control experiments using PVA nanofibers with PMS showed only 33.4% degradation, confirming that the incorporated cobalt oxide enhances PMS activation. Notably, PVA/Co_3_O_4_ nanofibers alone showed no significant TC adsorption or degradation, indicating that the degradation process is primarily driven by PMS activation.

A 2^2^ central composite factorial design was implemented to optimize degradation conditions, varying pH (3–9) and PMS concentration (0.567–1.700 mmol L^–1^) (Table 4). This pH range was selected based on the operational range of sulfate radicals generated through PMS activation.^43^ The PMS concentration range (0.567 to 1.700 mmol L^–1^) was chosen based on established literature precedents.^44^

**Table 4:** Central Composite Factorial Design 2^2^ Matrix with the Response Values for the Degradation of a 16.8 mg L^–1^ TC Solution using PVA/Co_3_O_4_ Non-woven in the Presence of PMS at the End of a 60 min Treatment

experiment	pH	PMS (mmol L^–1^)	degradation (%)
1	3.0	0.567	51.5
2	3.0	1.700	50.9
3	9.0	0.567	67.8
4	9.0	1.700	58.7
5	6.0	1.130	48.0
6	6.0	1.130	48.4
7	6.0	1.130	49.7

The highest degradation efficiency (67.8%) was achieved at pH 9 with a PMS concentration of 0.567 mmol L^–1^ (10-fold higher than TC’s molar concentration). Statistical analysis (F-test) confirmed that pH and PMS concentration significantly influence degradation efficiency (Figure 9 and Table S1). A basic pH in the reaction medium is advantageous, as acidic conditions can alter the crystallinity of the nanomaterial, lead to metal leaching into the solution, and cause changes in the morphological structure. These factors can result in a loss of catalytic performance and potential contamination of the reaction medium.^45^ Co_3_O_4_-based catalysts activate PMS through the formation of Co­(OH)^+^ as the active species, which forms more readily under neutral to slightly basic conditions. At pH 9, the equilibrium favors Co­(OH)^+^ formation, leading to enhanced PMS activation and sulfate radical generation (eqs 4 and 5).^31,46^

4 $${Co}^{2+}+H_{2}O⇌{CoOH}^{+}+H^{+}$$

5 $${CoOH}^{+}+{HSO}_{5}^{-}\to {CoO}^{+}+{\text{SO}}_{4}^{\cdot -}+H_{2}O$$

**Figure 9:**
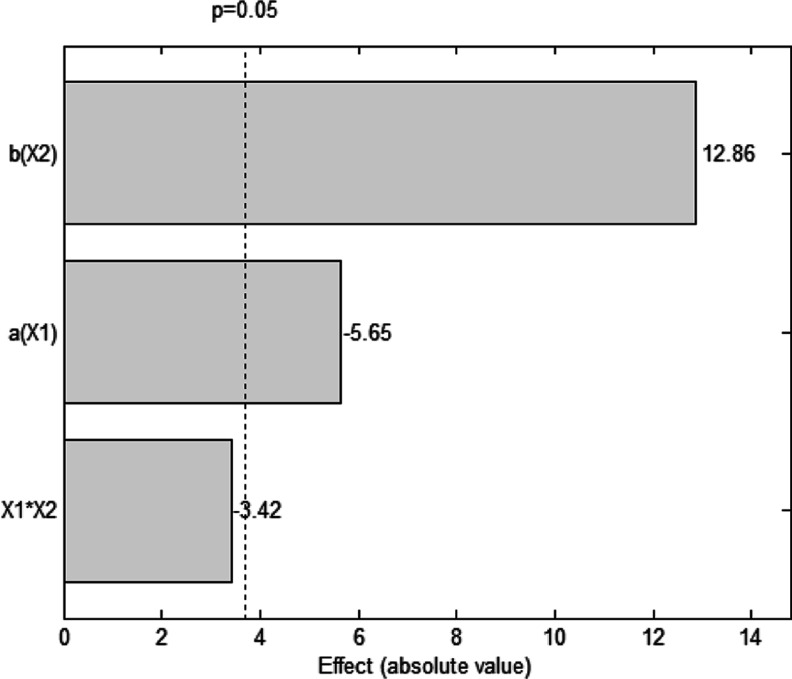
Pareto chart relating the significance of the variables pH and PMS concentration in the % degradation of tetracycline using nonwoven poly­(vinyl alcohol) incorporated with cobalt oxide (PVA/Co_3_O_4_). Conditions: 16.8 mg L^–1^ TC solution, under constant stirring.

Under acidic conditions, excess H^+^ ions compete with Co^2+^ for available PMS, reducing Co­(OH)^+^ formation and hindering activation. Additionally, H^+^ ions can scavenge both SO_4_^•–^ and ^•^OH radicals, further decreasing their availability for TC degradation (eqs 6,7).^43,47^

6 $${\text{SO}}_{4}^{\cdot -}+H^{+}\to {HSO}_{4}^{\cdot -}$$

7 OH·+H+→H2O

Enhanced PMS adsorption on the fiber surface near catalytic sites increases radical species generation. However, excessive PMS concentrations can lead to the self-extinction of sulfate radicals and the formation of persulfates, reducing treatment efficiency.^47,48^

Electrospun materials have emerged as promising catalysts for peroxymonosulfate (PMS) activation, primarily due to their high surface area and tunable properties. Previous studies have explored various cobalt-based electrospun catalysts, including Co_3_O_4_ nanofibers derived from polyvinylpyrrolidone (PVP),^49,50^ cobalt ferrite nanofibers from polyacrylonitrile (PAN),^50^ and cobalt ferrite nanoparticles supported on PAN-derived carbon nanofibers.^51^ While these earlier catalysts showed excellent performance (Table 5), they required calcination to remove the polymer matrix. Our PVA/Co_3_O_4_ composite nanofibers overcome two significant limitations: the need for high-temperature calcination and post-treatment catalyst separation. The Co_3_O_4_ nanoparticles are effectively immobilized within the biodegradable PVA matrix, maintaining accessible catalytic sites while preserving mechanical integrity and operational stability.

**Table 5:** Comparison of Cobalt-Based Catalysts for Organic Contaminant Degradation via PMS Activation

catalyst system	synthesis method	target pollutant	experimental conditions	removal efficiency	refs.
CONF (PVP–Co_3_O_4_)	electrospinning + calcination	acid red 27	PMS: 100 mg L^–1^; catalyst: 25 mg L^–1^; pH: 1–9; *t*: 15 min	100% decolorization	^49^
CFNF (Co/Fe-PAN)	electrospinning + calcination	sulfosalicylic acid	PMS: 150 mg L^–1^; catalyst: 100 mg L^–1^; *t*: 60 min	100% degradation	^50^
CF@CNF (Co/Fe_2_O_4_–PAN)	electrospinning + carbonization	amaranth	PMS: 200 mg L^–1^; catalyst: 50 mg L^–1^; *t*: 120 min	100% decolorization	^51^
Co_3_O_4_@CNF (nanofibers carbon)/Co_3_O_4_)	NP synthesis + calcination	methylene blue	PMS: 0.5 mmol L^–1^; catalyst: 100 mg L^–1^; *t*: 60 min	85% degradation	^52^
Co-CNFs (nanofibers carbon/Co)	electrospinning + carbonization	methylene blue; Evans blue; orange G	PMS: 100 μmol L^–1^; catalyst: 0.2 g L^–1^; *t*: 6–30 min	100% degradation	^53^

### Mechanism of Generation of Reactive Species

Figure 10 presents a mechanism for obtaining radical species using specific capture agents (scavengers). Scavengers can react with reactive species more rapidly than expected to react with the target contaminant (in this case, TC). Sodium azide captures singlet oxygen (^1^O_2_) radical species (*k* = 10^8^ M^–1^ s^–1^).^54,55^ Methanol exhibits similar reactivity between SO_4_^•–^ and ^•^OH (*k* = 1.0 × 10^7^ and 9.7 × 10^8^ M^–1^ s^–1^, respectively), causing the capture of these radical species.^56,57^ The *t*-butanol (TBA) is supposed to capture preferentially ^•^OH radicals (*k* = 3.8–7.6 × 10^8^ M^–1^ s^–1^).^58^

**Figure 10:**
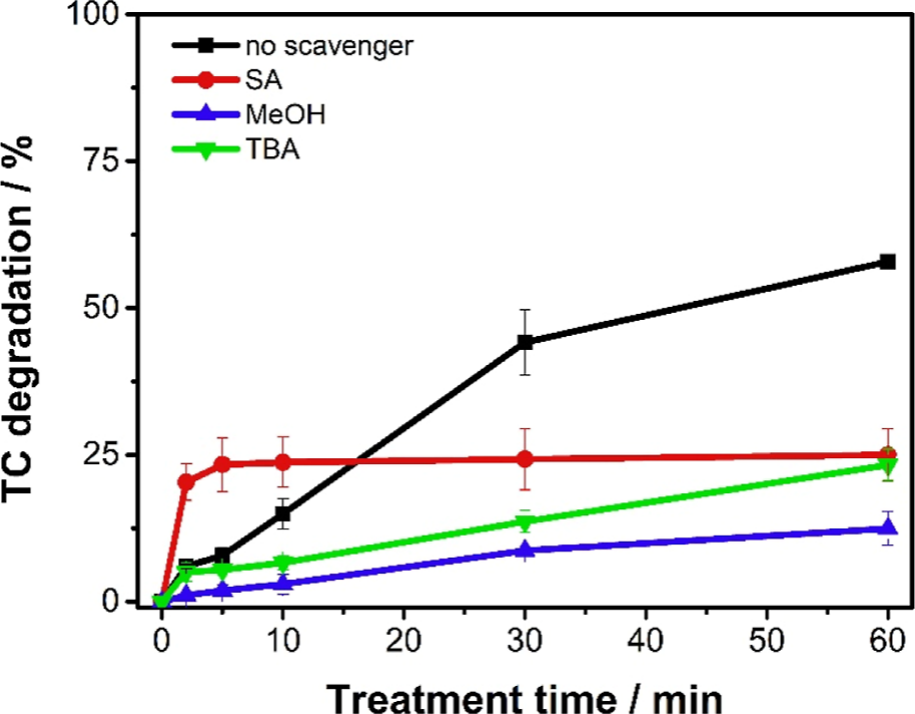
Influence of scavengers on the degradation of tetracycline hydrochloride by the PVA/Co_3_O_4_ + PMS system using sodium azide (SA), methyl alcohol (MeOH), and *tert*-butyl alcohol (TBA) in the proportion of 50 × [PMS] = 5.6 × 10^–2^ mol L^–1^. Conditions: 16.8 mg L^–1^ TC solution, pH 9, [PMS] = 0.567 mmol L^–1^, under constant stirring.

Without any scavenger, there is a degradation efficiency of 58.1% in 60 min of treatment. However, in the presence of sodium azide, there is a decrease to 21.7% degradation, indicating the inhibition of TC degradation by capturing singlet oxygen species.^59^

On the other hand, it is noted that when radical scavengers (methanol or *t*-butanol) are added, there is also inhibition of degradation, indicating that there is also a contribution from radical species.

A degradation curve with MeOH demonstrates 8.6% degradation, indicating the presence of radical species ^•^OH and SO_4_^•–^; when compared with TBA, 23.3% degradation is observed, meaning the higher percentage of degradation confirms the presence of SO_4_^•–^ radicals (which are not readily captured by TBA), indicating that both species can contribute to drug degradation.

Considering the degradation curves shown in Figure 10, zero-order kinetics were proposed for the MeOH and TBA scavengers. However, pseudo-first-order kinetics was observed for SA (NaN_3_), where the concentration curve behavior undergoes significant changes at 2, 5, and 10 min due to the reaction of the salt with TC, visually observed by the color change even in the absence of PMS. Thus, the rate constants were determined and are presented in Table 6. The contributions of radical and nonradical pathways to TC degradation through the PVA/Co_3_O_4_/PMS system were calculated using eqs 8 and 9.^60^

8 $$R_{nonradical}=\frac{k_{MeOH}}{k_{total}}\times 100\%$$

9 $$R_{radical}=100\%-R_{nonradical}$$

**Table 6:** Rate Constants (*k*) Obtained by Zero-Order Kinetics for the Degradation of 25 mL of TC (16.8 mg L^–1^) using PVA/Co_3_O_4_ + PMS in the Absence and Presence of Different Scavengers^a^

scavenger	species captured	*k* (mol L^–1^ min^–1^)	*R* ^2^	contribution (%)
-	-	0.987	0.892	-
MeOH	SO_4_^•–^ and ^•^OH	0.208	0.959	78.9
TBA	^•^OH	0.348	0.958	-
NaN_3_	^1^O_2_	-	-	21.1

a The radical and nonradical contributions to the mechanism of generation of reactive species were calculated using eqs 8 and 9. *k* – rate constant, *R* ^2^ – Coefficient of determination. Contribution of radical and nonradical pathways in the PVA/Co_3_O_4_/PMS degradation system in the presence of scavengers.

Where *R*_nonradical_ represents the contribution of the singlet oxygen generation mechanism, and *R*_radical_ represents the contribution of the radical species generation mechanism; *k*_MeOH_ is the rate constant in the presence of methanol, and *k*_total_ is the rate constant in the absence of any scavenger.

Based on the scavenger experiments, a mechanism for activating PMS by cobalt in the PVA/Co_3_O_4_ system can be proposed. The contribution of 78.9% of radicals identifies them as the principal active species formed in the system. To maintain reactive species generation in the system, forming CoOH^+^ species is essential and is the limiting step for generating SO_4_^•–^ radicals (eq 4). The regeneration of Co^2+^ by reducing Co^3+^ ions occurs through the consumption of an HSO_5_^–^ ion (PMS), allowing for the succession of the reaction at low catalyst concentration (eq 5, 10 and 11).^61^ PMS activation occurs through both radical (predominant) and nonradical pathways, as evidenced in Table 6. While PMS activation does not directly generate hydroxyl radicals, these may be generated by the reaction of sulfate radicals with H_2_O or OH^–^ ions (eqs 13 and 14). The nonradical pathway, estimated to contribute 21.1% to TC degradation, involves singlet oxygen (^1^O_2_) generation.^62^ The complete mechanism includes several interconnected pathways (eqs 10–17).^63,64^ Initially, the activation of PMS by Co^2+^ leads to the formation of sulfate radicals (SO_4_^•–^). Subsequently, a series of reactions generates various reactive oxygen species, including hydroperoxyl radicals (HO_2_^•^) (eq 15), which can dissociate to form superoxide radicals (O_2_^•–^) (eq 16). These superoxide radicals can further react with hydroxyl radicals to generate singlet oxygen, ^1^O_2_ (eqs 17 and 18), contributing to the nonradical oxidation pathway. This comprehensive mechanism, depicted in Figure 11, explains the observed distribution between radical and nonradical degradation pathways in our system. The key reactions in this process are

10 $${CoO}^{+}+{2H}^{+}⇆{Co}^{3+}+H_{2}O$$

11 $${Co}^{3+}+{HSO}_{5}^{-}\to {Co}^{2+}+{\text{SO}}_{5}^{\cdot -}+H^{+}$$

12 $${\text{SO}}_{4}^{\cdot -}+organics\to \left[intermediateproducts\right]\to {CO}_{2}+H_{2}O$$

13 $${\text{SO}}_{4}^{\cdot -}+H_{2}O\to^{\cdot }OH+{\text{SO}}_{4}^{\cdot -}+H^{+}$$

14 $${\text{SO}}_{4}^{\cdot -}+{OH}^{-}\to^{\cdot }OH+{\text{SO}}_{4}^{2-}$$

15 OH·+H2O2→HO2·+H2O

16 $${HO}_{2}^{\cdot }\to H^{+}+O_{2}^{\cdot -}$$

OH·+O2·−→O12+OH− 17

2O2·−+2H+→O12+H2O2 18

**Figure 11:**
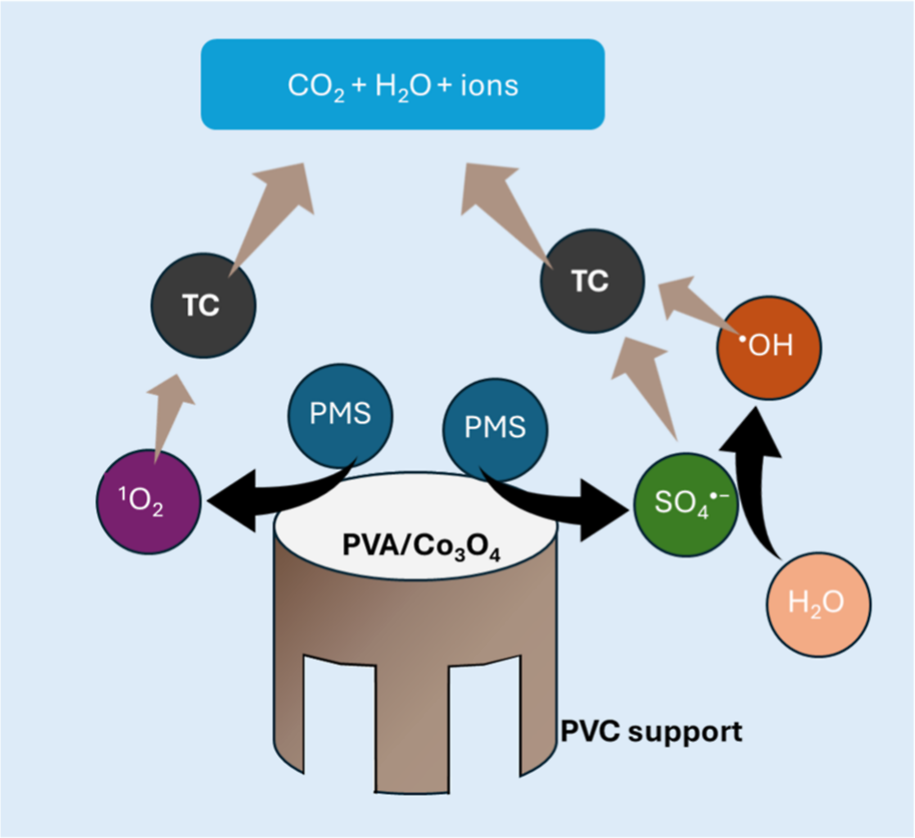
A proposed mechanism for the system containing the electrospun PVA/Co_3_O_4_ nanomaterial activating PMS to generate reactive species via radical (^•^OH and SO_4_^•–^) and nonradical (^1^O_2_) pathways for tetracycline (TC) degradation.

### Stability of Electrospun PVA/Co_3_O_4_

Figure 12 demonstrates the reusability of the nanomaterial across multiple degradation cycles, confirming its sustained efficiency in generating SO_4_^•–^ and ^•^OH radical species without performance loss due to catalyst poisoning or cobalt degradation. The initial degradation efficiency of 33.5% remained consistent throughout subsequent cycles, with the membrane maintaining its catalytic performance. EDX analyses of the treated solution confirmed the absence of leached cobalt oxide, validating the effectiveness of the cross-linking process. This cross-linking provides enhanced aqueous stability to the membrane while preserving the accessibility of cobalt-containing catalytic sites.

**Figure 12:**
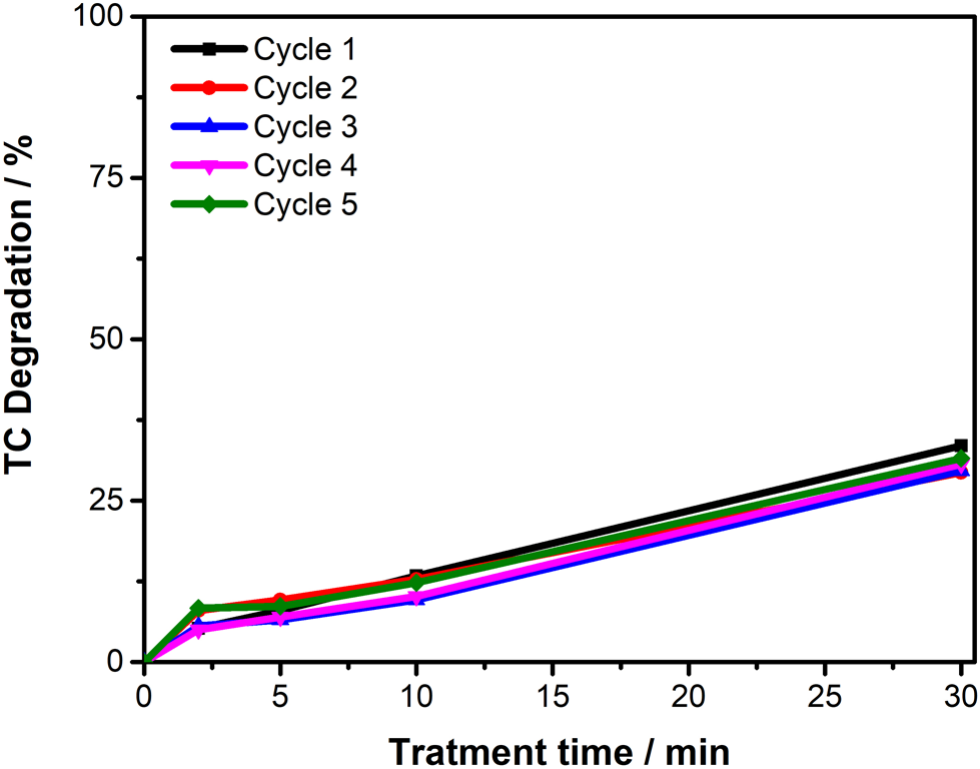
Reusability analysis of the PVA/Co_3_O_4_ nanomaterial showing TC degradation efficiency over five consecutive degradation cycles. Conditions: 16.8 mg L^–1^ TC solution, pH 9, [PMS] = 0.567 mmol L^–1^, under constant stirring.

## Conclusions

Electrospinning was successfully employed to fabricate PVA nanofibers incorporating Co_3_O_4_, resulting in a novel catalytic material for peroxymonosulfate (PMS) activation. Using PVA, a cheap, water-soluble, and biodegradable polymer, aligns with the principles of green chemistry and sustainable materials development. Incorporating Co_3_O_4_ induced significant structural changes in the nanofibers, enhancing their crystallinity and mechanical properties, which are crucial for practical applications in water treatment. The PVA/Co_3_O_4_ nanofibers demonstrated efficient catalytic activation of PMS, predominantly through a radical pathway (78.9%) involving SO_4_^•–^ and ^•^OH radicals, with a minor contribution from nonradical ^1^O_2_ (21.1%). This activation led to the successful degradation of tetracycline (TC), a model antibiotic contaminant. Under optimized conditions (pH 9, [PMS] = 10×[TC]), the system achieved a degradation efficiency of 67.8%. The results highlight the potential of Co_3_O_4‑_incorporated PVA nanofibers as sustainable catalysts for PMS activation in water treatment applications. The enhanced properties of the nanofibers, combined with the dual radical and nonradical degradation pathways, make this system a promising candidate for removing various organic pollutants. Future research will mainly investigate the degradation of other emerging contaminants, the long-term stability and reusability of the catalyst, and explore the potential for real-world water treatment applications.

## Supplementary Material



## Notes

Additional graphs of materials characterization, such as SEM, EDX, DRX, TGA, and the F-test for the Central composite factorial design 2^2^ (PDF)
