# Supplementary material for: Electrospun PVA/Co3O4 Nanofibers: A Sustainable Catalyst for Peroxymonosulfate-Mediated Degradation of Tetracycline
Source: ACS Omega. 2025 Jun 25;10(26):27883–93. doi: 10.1021/acsomega.5c01013 (PMC12242669; doi:10.1021/acsomega.5c01013)
Supplement: Supplementary file 1 [file ao5c01013_si_001.pdf]

SUPPORTING INFORMATION

Electrospun PVA/Co_3O_4 Nanofibers: A Sustainable Catalyst for Peroxymonosulfate-Mediated Degradation of Tetracycline

Felipe G. Kirchhoff [†], *Gabriel N. Fraga* [†,‡], *Reinaldo A. Bariccatti* [†], *Douglas C. Dragunski* [†], *Guilherme G. Bessegato* [§]*

[†] Universidade Estadual do Oeste do Paraná (UNIOESTE), Rua da Faculdade 645, 85903-000 Toledo, PR, Brazil

[‡] Department of Chemistry, Universidade Estadual de Maringá (UEM), Av. Colombo, 5790 - Zona 7, 87020-900 Maringá, PR, Brazil

[§] Universidade Tecnológica Federal do Paraná (UTFPR), Dois Vizinhos Campus, Estrada para Boa Esperança, km 04, 85660-000 Dois Vizinhos, PR, Brazil

*Corresponding author. E-mail address: bessegato@utfpr.edu.br (G.G. Bessegato).

*Phone number: +554635368209

MATERIALS CHARACTERIZATION

Scanning Electron Microscopy of Co_3O_4

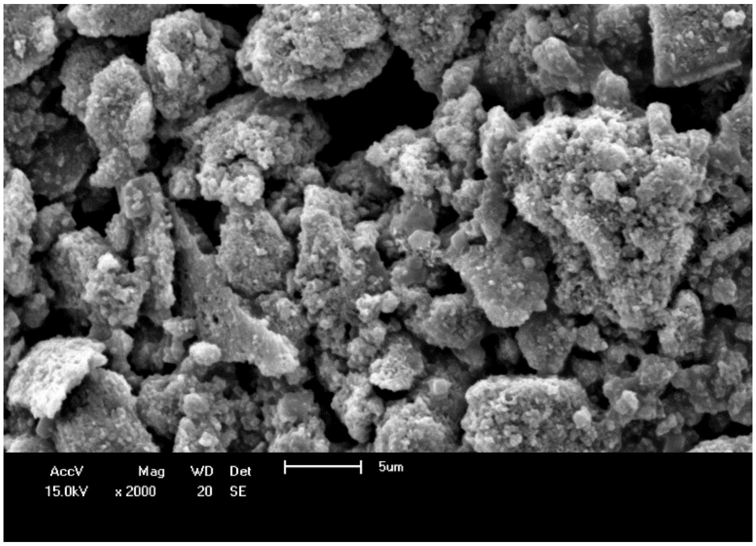

Figure S1. Scanning electron microscopy (SEM) image of the synthesized cobalt oxide crystals at a magnification of 2000x.

X-Ray Diffraction of Co_3O_4

Sample holder

Figure S2 presents the XRD spectrum obtained from the sample holder. The distinct signals observed are attributed to the thickness of the electrospun nanomaterial. During analyses with non-woven fabric samples, the X-ray beams penetrate the sample itself and the underlying device holder, resulting in the detection of signals from both.

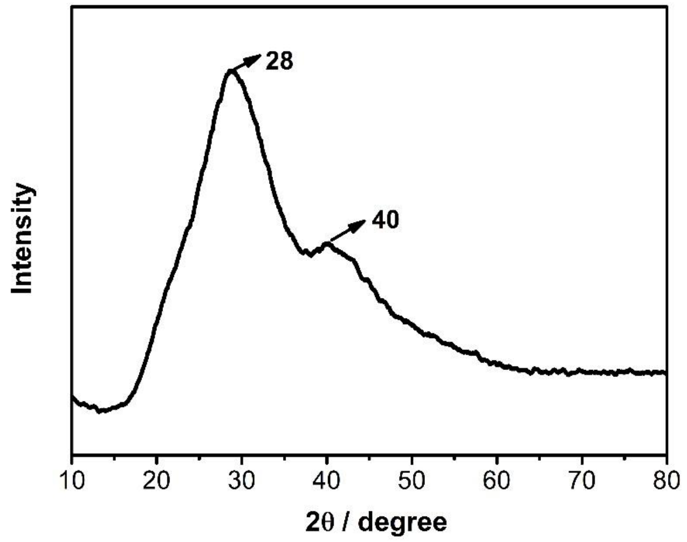

Figure S2. X-ray diffraction analysis of the support apparatus used for the analyses of PVA materials

Figure S3 presents the XRD spectra obtained from the synthesized cobalt oxide. The observed peaks were indexed (JCPDS card No. 01-071-0816), and the synthesized product was characterized as cobalt oxide, Co_3O_4.

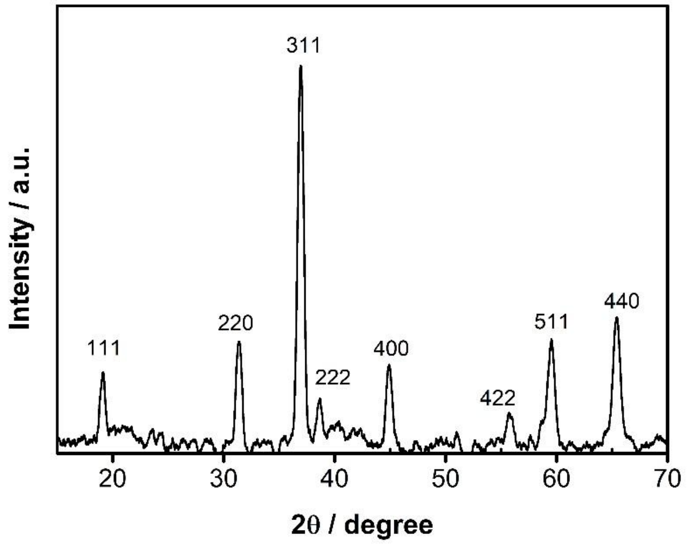

Figure S3. X-ray diffraction (XRD) spectra of the synthesized cobalt oxide.

Energy Dispersive X-ray Fluorescence analysis (EDX)

Figure S4 presents the EDX report for electrospun non-woven PVA fibers (without cobalt oxide), revealing a negligible percentage of elements other than carbon.

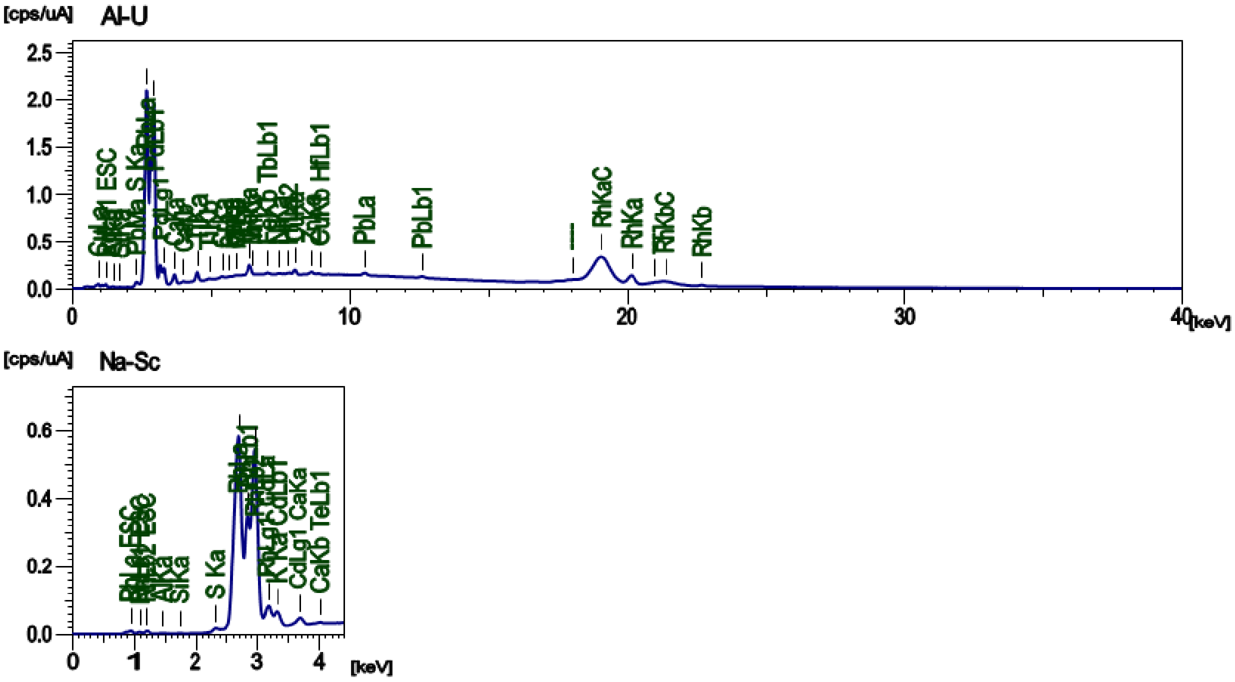

Quantitative Result							
Analyte	Result			Std.Dev.	Calc.Proc	Line	Intensity
Al	0.102	%		[0.020]	Quan-FP	AlKa	0.0095
K	0.032	%		[0.001]	Quan-FP	K Ka	0.2913
Si	0.020	%		[0.003]	Quan-FP	SiKa	0.0108
Ca	0.017	%		[0.001]	Quan-FP	CaKa	0.2355
S	0.016	%		[0.001]	Quan-FP	S Ka	0.0776
Ti	0.010	%		[0.000]	Quan-FP	TiKa	0.6050
Fe	0.008	%		[0.000]	Quan-FP	FeKa	0.8874
Pb	0.007	%		[0.001]	Quan-FP	PbLb1	0.1909
Sm	0.004	%		[0.000]	Quan-FP	SmLa	0.1062
Cu	0.004	%		[0.000]	Quan-FP	CuKa	0.4163
Zn	0.002	%		[0.000]	Quan-FP	ZnKa	0.2564
Cr	0.002	%		[0.000]	Quan-FP	CrKa	0.1499
Mn	0.002	%		[0.000]	Quan-FP	MnKa	0.1610
Ni	0.001	%		[0.000]	Quan-FP	NiKa	0.1612
C	99.773	%		[------]	Balance	--------	--------

Figure S4. EDX report for electrospun non-wovens of PVA.

However, Figure S5 presents the EDX report for electrospun non-woven PVA/Co_3O_4 material. It indicates that cobalt is present in a significant percentage, distinguishing it from the noise signal of the other elements also shown in Figure S4.

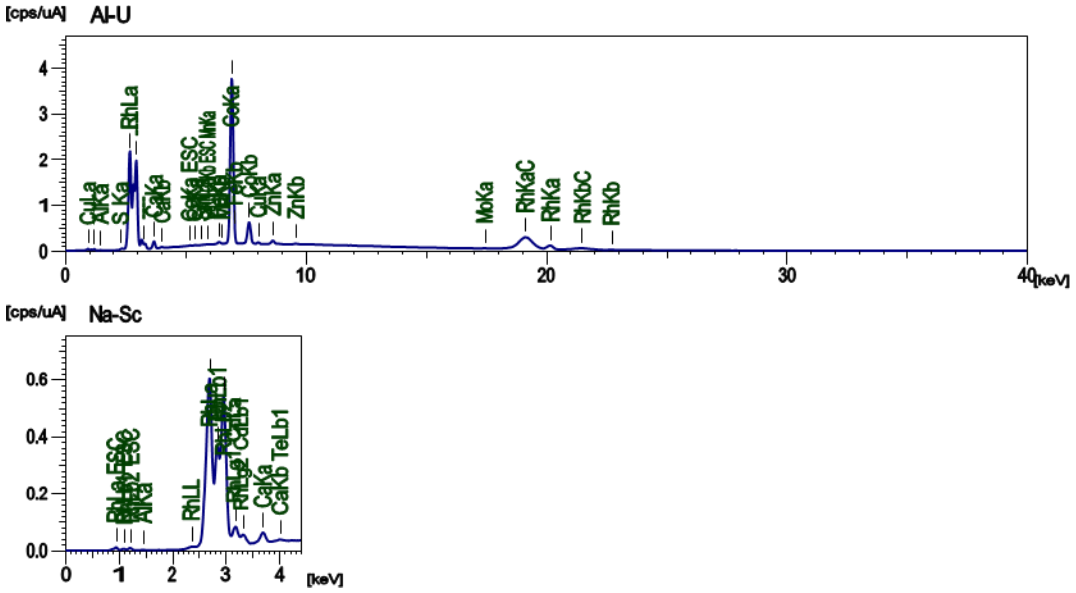

Quantitative Result								
Analyte	Result			Std.Dev.	Calc.Proc	Line	Intensity	
Co	0.308	%		[0.001]	Quan-FP	CoKa	27.2908	
Al	0.101	%		[0.021]	Quan-FP	AlKa	0.0089	
Ca	0.036	%		[0.001]	Quan-FP	CaKa	0.4202	
S	0.022	%		[0.001]	Quan-FP	S Ka	0.1903	
Zn	0.008	%		[0.000]	Quan-FP	ZnKa	0.6933	
Fe	0.004	%		[0.000]	Quan-FP	FeKa	0.3996	
Sm	0.004	%		[0.001]	Quan-FP	SmLa	0.0931	
Cu	0.003	%		[0.000]	Quan-FP	CuKa	0.2785	
Cr	0.002	%		[0.000]	Quan-FP	CrKa	0.1394	
Mo	0.002	%		[0.000]	Quan-FP	MoKa	0.0700	
Mn	0.002	%		[0.000]	Quan-FP	MnKa	0.1190	
C	99.509	%		[------]	Balance	--------	--------	

Figure S5. EDX report for electrospun non-wovens of PVA/Co_3O_4 material.

Thermogravimetric analysis (TGA) and differential thermogravimetric analysis (DTG) of Co_3O_4

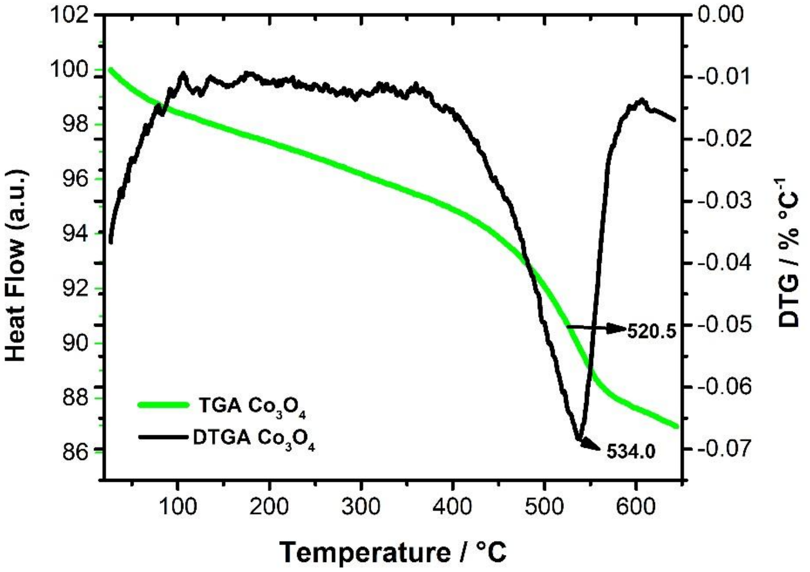

Figure S6. Thermogravimetric analysis (TGA) and differential thermogravimetric analysis (dTGA) curves of the synthesized cobalt oxide.

MATHEMATICAL DECONVOLUTION OF THE TGA

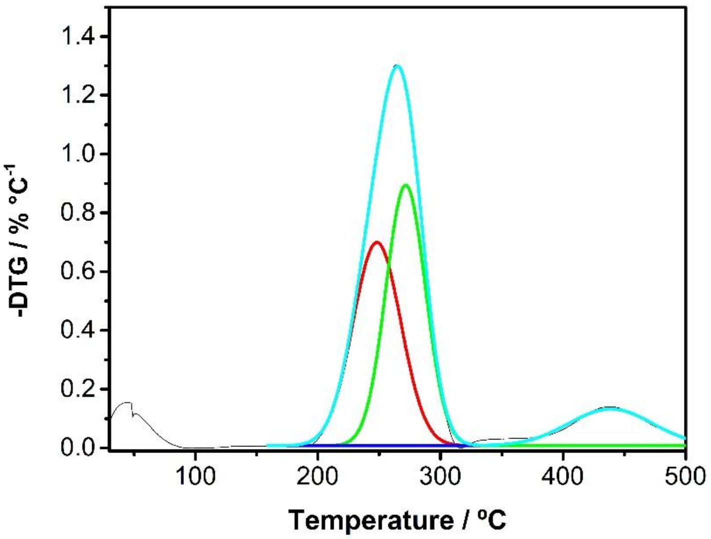

Figure S7. Mathematical deconvolution of the TGA analyses of the polyvinyl alcohol (PVA) membrane without the crosslinking process.

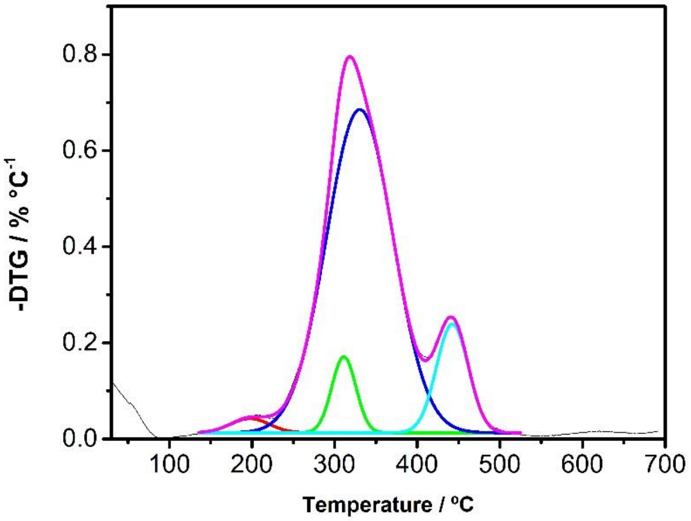

Figure S8. Mathematical deconvolution of the TGA analyses of the crosslinked polyvinyl alcohol membrane reticulated with citric acid.

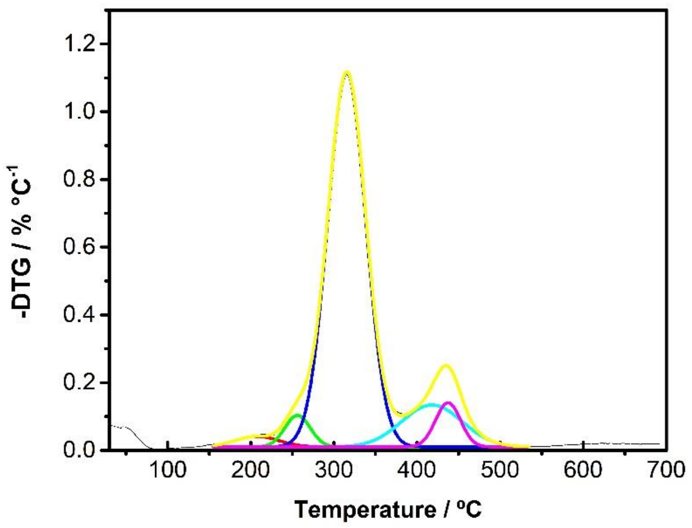

Figure S9. Mathematical deconvolution of the TGA analyses of the polyvinyl alcohol (PVA) membrane incorporated with cobalt oxide (5g L−1) (PVA/Co_3O_4/RET).

Central composite factorial design 2^2

Table S1 presents the mean values and variance for each of the variables analyzed in the experimental design (pH and [PMS]). Analysis by the F-test did not reject the null hypothesis, indicating homogeneity of the data within the experimental design.

Table S1. F-test: two-sample for variances presented.

	Variable 1	Variable 2
Mean	48.71	53.535
Variance	0.7396	0.00845
Observations	3	2
gl	2	1
F	87.52663	
P(F<=f) uni-caudal	0.075366	
F critical uni-caudal	199.5	
